# Characterization of the Portuguese population diagnosed with retinoblastoma

**DOI:** 10.1038/s41598-022-08326-6

**Published:** 2022-03-14

**Authors:** Guilherme Castela, Joana Providência, Madalena Monteiro, Sonia Silva, Manuel Brito, Joaquim Sá, Barbara Oliveiros, Joaquim Neto Murta, Zelia Correa, Miguel Castelo Branco

**Affiliations:** 1 Department of Ophthalmology, Centro Hospitalar e Universitário de Coimbra, Praceta Professor Mota Pinto, 3004-561 Coimbra, Portugal; 2 Faculty of Medicine, University of Coimbra, Azinhaga de Santa Comba, 3000-548 Coimbra, Portugal; 3 Coimbra Institute for Biomedical Imaging and Translational Research (CIBIT), Azinhaga de Santa Comba, 3000-548 Coimbra, Portugal; 4 Department of Pediatric Oncology, Centro Hospitalar e Universitário de Coimbra, Coimbra, Portugal; 5 Medical Genetics Unit, Centro Hospitalar e Universitário de Coimbra, Coimbra, Portugal; 6 Bascon Palmer Eye Institute, University of Miami, Miami, FL USA

**Keywords:** Cancer, Eye diseases, Cancer, Cancer epidemiology, Cancer genetics, Eye cancer

## Abstract

The purpose of this study is to characterize demographically and genetically the Portuguese population with retinoblastoma; to report the clinical stage at presentation and its impact on survival and ocular preservation rate and, finally, to assess the incidence of retinoblastoma in Portugal. Retrospective observational study including children consecutively diagnosed with retinoblastoma at the Portuguese National Referral Center of Intraocular Tumors, between October 2015 and October 2020. Twenty-eight children were diagnosed with retinoblastoma at our center, 15 hereditary from which 12 presented with bilateral retinoblastoma and 3 were unilateral. The overall mean age at diagnosis was 13.6 ± 11.1 months with hereditary retinoblastomas diagnosed slightly earlier at 9.6 ± 6.3 months. A familial history of retinoblastoma was found in only 4 (14.3%) of the cases. A pathogenic mutation in the *RB1* gene was found in 13 (46.4%) of the children. The most frequent sign at referral was leukocoria in 71.4% of patients. Considering the ICRB classification of the tumors, 84.6% of non-hereditable hereditary retinoblastomas were referred to our center in advanced stages. In the group of hereditable retinoblastomas 86.7% presented with one of the eyes with advanced intraocular retinoblastoma. Fourteen children had one eye enucleated due to retinoblastoma. No deaths were registered during the study period. Considering the incidence analysis, we registered a year-of-birth controlled incidence analysis of 4.04 per 100.000 living births (IC 95% 1.59–6.49). This is the first characterization of the Portuguese Population diagnosed with Retinoblastoma in the National Reference Center.

## Introduction

Retinoblastoma is the most prevalent intraocular malignancy in the childhood, with an estimated incidence of one out of 16.000–18.000 live births worldwide, based on epidemiological studies from the United States and Northern Europe^1–3^. Its incidence is similar across populations and does not vary according to gender, ethnicity, or socio-economic status. However, the clinical stage at presentation of the disease, which has a major impact on survival and ocular preservation rate, significantly differs among patients from low-income and high-income countries. Furthermore, according to a recently published multicenter study regarding retinoblastoma diagnosis in 153 countries, lower-income countries were associated with older presentation age, higher proportion of locally advanced disease and distance metastasis and a smaller proportion of familial history of Retinoblastoma and of bilateral hereditable retinoblastoma (23.5 vs. 35.4%, respectively), when comparing to high-income countries^4^.

Retinoblastoma originates mostly in association with a mutation in the *RB1* gene, a tumor suppression gene. The mutation can be hereditary, associated with hereditary retinoblastoma, an autosomal dominant susceptibility for retinoblastoma, or can occur sporadically in a somatic retinal cell. Hereditary retinoblastoma is associated with bilateral and multifocal disease. However, it is estimated that 10–20% of children with unilateral retinoblastoma have a germline mutation, making genetic counseling and testing compulsory to all the children diagnosed with retinoblastoma. Furthermore, most hereditary retinoblastoma patients do not have a family history of retinoblastoma. This can be explained with mutations occurring de novo at or soon after conception, which, depending on the stage of development at which the mutation occurs, can affect only some of the fetus’ cells, resulting in a mosaicism with more unpredictable hereditary behavior^5,6^. Some Retinoblastoma do not show inactivation of RB1 gene, but amplification of the MYCN gene, associated with especially young age at presentation and large and invasive tumors^7^.

The true incidence, demographic features, and clinical characteristics at presentation in the Portuguese population have not been described in the past. Since October 2015, Portuguese children diagnosed with retinoblastoma started to be treated in the Portuguese Referral Center for the Management of Intraocular Tumors. The center integrates a multidisciplinary team providing all the treatment modalities available for the management of retinoblastoma, including intra-arterial chemotherapy, systemic intravenous chemotherapy, intravitreal chemotherapy, cryotherapy, transpupillary thermotherapy, brachytherapy, and external beam radiation therapy, and is currently the only center in Portugal treating children diagnosed with retinoblastoma. When other centers suspect or diagnose retinoblastoma patients, an immediate referral is made to the National Reference Center, creating the ideal conditions for the characterization of the Portuguese population affected with this condition.

The aim of this study is to (1) characterize demographically and genetically the Portuguese population with retinoblastoma; (2) to report the clinical stage at presentation and its impact on survival and ocular preservation rate and, finally, to (3) assess the incidence of retinoblastoma in Portugal.

## Methods

### Study population

We performed a retrospective observational study including children consecutively diagnosed with retinoblastoma at the Portuguese National Referral Center of Intraocular Tumors, between October 2015 and October 2020. Our study was approved by the ethics committee of University of Coimbra and of Centro Hospitalar e Universitário de Coimbra and was performed according to the tenets of Declaration of Helsinki.

Inclusion criteria was the diagnosis of retinoblastoma in children referred to the Portuguese National Reference Center of Intraocular Tumors. Exclusion criteria included children born in Portuguese-speaking African countries evacuated from their hometown country for the management of retinoblastoma at our center.

At the diagnosis, the child’s complete medical history was obtained, and ophthalmological examination performed by a team specialized in pediatric ophthalmology and ocular oncology. Examination under anesthesia was performed for detailed fundus evaluation and to capture and record fundus images with RetCam. All the children were also observed by a team of pediatric oncologists for a complete comprehensive systemic evaluation. Staging was performed by the multidisciplinary team according to the International Classification of Retinoblastoma (ICRB) staging system^8^. Blood samples were collected to all the participants and were used to obtain a complete genetic testing for *RB1* mutations. At baseline and periodically during the follow up (every 3–6 months), the included participants underwent cerebral magnetic resonance imaging (MRI) for evaluation of pineal involvement.

Patients with bilateral retinoblastoma, pineal involvement, positive family history of retinoblastoma or with a *RB1* pathogenic variant found in the genetic testing were included in the hereditary retinoblastoma group. Remaining patients (unilateral retinoblastomas with negative family history and negative genetic testing) were included in the non-hereditary retinoblastoma group.

Clinical data was obtained from the clinical records of the patients and was used to perform the final database that was validated by two authors (GC and JP) before data analysis. Informed consent of all children was obtained from a parent and/or legal guardian.

### Statistical analysis

Patients were characterized in terms of demographics (region of birth, sex, age at diagnosis, clinical signs at presentation and familial history of retinoblastoma) and tumor characteristics (eye stage according to ICRB, multifocality and laterality).

Portuguese epidemiological data, namely annual living-births and population under 5 years-old, was obtained from the databases of the Portuguese National Statistics Institute (INE—Instituto Nacional de Estatística) and was used to calculate the Portuguese annual incidence. Incidence was calculated by standard analysis per children younger than 5 years of age (dividing the number of children diagnosed with retinoblastoma by the number of children younger than 5 years in the corresponding year) and per live births (dividing the number of children diagnosed with retinoblastoma by the number of live-born children in the corresponding year) and by birth cohort analysis. Birth cohort analysis is calculated after grouping the children diagnosed with retinoblastoma by year of birth and dividing this number by the live-births in the corresponding year. Standard annual analysis is highly susceptible to the age at diagnosis, and therefore, calculation based on birth cohorts is advised, rather than based on year of diagnosis^3^.

All statistical analysis were performed using IBM SPSS Statistics 26 for Windows. Chi-square tests were used for categorical variables and the non-parametric Mann–Whitney to continuous variables. The incident 95% confidence interval was calculated based on a Poisson distribution. For all the comparisons, a p-value under 0.05 was considered as statistically significant.

### Ethical approval

The ethics committee of Faculty of Medicine, University of Coimbra, approved this study.

### Consent to participate

Informed consent of all included participants was obtained from a parent and/or legal guardian.

## Results

### Demographic characterization of the Portuguese population

During the 5 years of study period, 28 children were diagnosed with retinoblastoma at our center. Fifteen children were included in the hereditary retinoblastoma group, from which 12 presented with bilateral retinoblastoma and 3 were unilateral but had a positive genetic test for a *RB1* pathogenic variant (2 of these had known family history of retinoblastoma).

The overall mean age at diagnosis was 13.6 ± 11.1 months. Hereditary retinoblastomas were diagnosed slightly precociously at 9.6 ± 6.3 months, compared to non-hereditary retinoblastomas in which the mean age at diagnosis was 18.3 ± 13.6 months (*p* = 0.065). A familial history of retinoblastoma was found in only 4 (14.3%) of the cases. Full demographic characteristics of the included children can be found in Table 1.

**Table 1:** Demographic and clinical characterization of the Portuguese population diagnosed with Retinoblastoma.

	Non-Hereditary Rb n = 13	Hereditary Rb n = 15	p-value	Total N = 28
Males (%)	6 (46.2)	8 (53.3)	0.705^1^	14 (50)
Age at diagnosis, mean (sd)	18.31 (13.63)	9.60 (6.32)	0.065^1^	13.64 (11.08)
Family History	–	4 (26.7)	–	4 (14.3)
Bilateral (%)	–	12 (80.0)	–	12 (42.9)
Pineal (%)	–	0 (0)	–	0 (0)
**Presenting symptom (%)**				
Leukocoria	11 (84.6)	9 (60.0)	0.150^1^	20 (71.4)
Strabismus	6 (46.2)	6 (40.0)	0.743^1^	12 (42.9)
Buphthalmus	0 (0)	2 (13.3)	0.172^1^	2 (7.1)
Cellulitis	0 (0)	2 (13.3)	0.172^1^	2 (7.1)
No symptoms	0 (0)	2 (13.3)	0.172^1^	2 (7.1)
**Geographic distribution**			0.061^1^	
North	0	5	–	5
Center	1	3	–	4
South	11	6	–	17
Islands	1	1	–	2

A pathogenic mutation in the *RB1* gene was found in 13 (46.4%) of the children. The mutations found in our cohort are represented in Table 2. In two children with bilateral disease, both with a negative familial history, a mutation for the *RB1* gene could not be found in the standard genetic testing and further testing is being performed using the pathology specimen.

**Table 2:** Mutations found in genetic testing.

RB1 mutations
c.1467C > A p.(Cys489Ter)
c.1072C > T p.(Arg358Ter)
c.1654C > T p.(Arg552Ter)
c.297G > A p.(Trp99Ter)
c.751C > T p.(Arg251Ter)
c.1450_1451del p.(Met484Valfs*8)
c.1128-2A > G p.?
c.(?_-1)_(1969 + 1_1961-1)
c.106del p.(Asp36Thrfs*29)
c.1947del p.(Tyr651Ilefs*7)
c.1023_1024del p.(Lys341Asnfs*5)
del *RB1*

Considering the geographic distribution of the included cases, 5 children were born in the north of the country, 4 in the center, 17 in the south and 2 in the Portuguese islands, but the age at diagnosis was not significantly different between the regions (11.9 months in the north, 16.5 months in the center, 14.76 in the south and 14.75 in the islands, *p* = 0.859). We considered four main regions of distribution, including mainland and islands, according to the existence of tertiary care hospitals.

### Clinical characterization at presentation and ocular and global survival analysis

At the time of the first observation at our center, we found that 71.4% of the cases had been referred due to leukocoria, the most common initial presentation symptom in our population, followed by strabismus that was present in 42.9% of the included participants (Table 1). Four children, all included in the hereditary retinoblastoma group, presented with advanced intraocular retinoblastoma manifesting with cellulitis (n = 2) and buphthalmus (n = 2). During the study inclusion period, no cases of extra-ocular retinoblastoma or pineal involvement were referred to our center. Two children with a previously known positive family history of retinoblastoma and a prenatal diagnosis of retinoblastoma were referred during their first months of life while still asymptomatic.

Considering the Classification of the tumors according to the ICRB (Table 3), non-hereditable hereditary retinoblastomas were referred to our center 2 (15.4%) in an early stage (group C) and the remaining in advanced intraocular stages (8 [61.5%] group D and 3 [23.1%] group E). Considering the hereditable retinoblastomas (Table 3), 13 (86.7%) presented with one of the eyes with advanced intraocular retinoblastoma (group D or E), from which 2 (13.3%) presented group D or E lesions in both eyes. In this group, 3 patients presented with unilateral retinoblastoma, staged as follows: 1 group A and 2 group D.

**Table 3:** Classification of non-hereditable and hereditable retinoblastoma according to International classification of Retinoblastoma (ICRB)^8^.

	Non-hereditable Rb n = 13	Hereditable Rb n = 15				
		A	C	D	E	Total
No Rb	–	1 (6.7)	–	2 (13.3)	–	3 (20)
A	–	–	1 (6.7)	1 (6.7)	–	2 (13.3)
B	–	–	–	2 (13.3)	2 (13.3)	4 (26.7)
C	2 (15.4)	–	–	2 (13.3)	2 (13.3)	4 (26.7)
D	8 (61.5)	–	–	1 (6.7)	1 (6.7)	2 (13.3)
E	3 (23.1)	–	–	–	–	–
Total	13 (100)	1 (6.7)	1 (6.7)	8 (53.3)	5 (33.3)	15 (100)

During the study period, 14 children had one eye enucleated due to retinoblastoma. In the non-hereditable retinoblastoma group 6 (46.2%) of the affected eyes were preserved. In the hereditary retinoblastoma group, 8 (53.3%) children had both eyes preserved and 7 (46.7%) children had one eye preserved. No deaths were registered during the study period (Table 4).

**Table 4:** Summary of the clinical management of Retinoblastoma patients.

	Non- Hereditary n = 13 eyes	Hereditary n = 27 eyes	Total N = 40 eyes
**First-line treatment, n (%)**			
Enucleation	2 (15.4)	3 (11.1)	5 (12.5)
Systemic chemotherapy	1 (7.7)	15 (55.6)	16 (40.0)
Intraarterial chemotherapy	10 (76.9)	8 (29.6)	18 (45.0)
**Additional treatments, n (%)**			
Local cryotherapy	0	2 (7.4)	2 (5.0)
Transpupilary thermotherapy	5 (38.5)	20 (74.1)	25 (62.5)
Intravitreal chemotherapy	1 (7.7)	1 (3.7)	2 (5.0)
Enucleation during follow-up, n (%)	5 (38.5)	4 (14.8)	9
**Patient status at final visit, n (%)**			
Survival	13 (100)	28 (100)	40 (100)
Enucleation	7 (53.8)	7 (25.9)	14 (35.0)
Recurrences	2 (15.4)	6 (22.2)	8 (20)

### Retinoblastoma incidence in the Portuguese population

Considering the standard incidence analysis, we registered a retinoblastoma annual incidence rate of 1.05 per 100.000 children under the age of 5 years (IC 95% − 0.00 to 2.09) and of 5.36 per 100.000 living births (IC 95% 0.11–10.62). Year-of-birth controlled analysis registered an annual incidence of 4.04 per 100.000 living births (IC 95% 1.59–6.49).

## Discussion

We report the first clinical and genetic characterization of the Portuguese population diagnosed with retinoblastoma, based on the first 5 years of experience of the Portuguese national referral center for the treatment of retinoblastoma. During the study period, 28 children were diagnosed with retinoblastoma and managed at our center. The mean age at the time of diagnosis in our population was 13.64 ± 11.08 months, which, considering the mean age of 14.1 months registered in high-income countries, reported recently in a global analysis by national income level^4^, represents a considerably good time of referral and diagnosis. Despite the age of diagnosis being similar to other high-income countries, and the fact that no cases of extraocular involvement were referred to our center, a considerable proportion of patients were referred with advanced intraocular disease (84.6% of non-hereditable retinoblastoma cases and 86.7% of hereditable retinoblastoma presented with at least one eye group D or E according to ICRB). In the future, we hope to continue to increase the awareness of this disease among general practitioners, pediatricians, parents, and school workers in order to attempt earlier diagnosis at our country. Various factors can contribute to a delay in retinoblastoma diagnosis, which is related to a worse prognosis. These factors include the lack of awareness about retinoblastoma in general population, lack of national screening program and lack of training in screening by primary health care workers and pediatricians^9^.The difference at the age of diagnosis between hereditable and non-hereditable retinoblastomas, also reported in other low and high income countries^4^, is in our case partially explained by the earlier referral to our center in children with a known familial history of retinoblastoma, allied with the earlier clinical manifestations in hereditary cases.

The symptoms leading to referral in Portuguese patients were also similar to previous literature reports, in which leukocoria is usually the most common first symptom of disease, followed by strabismus. Four children (corresponding to 14.3% of the sample) presented with advanced intra-ocular disease (cellulitis or buphthalmus), which is superior to reports from high-income levels where the presence of orbital cellulitis at presentation was only 0.8%^4^. Globe preservation is one of the most important treatment goals in the present and we hope that, in the following years, combining a patients’ referral at earlier disease stages with a greater experience with intra-arterial chemotherapy and other globe-preserving treatment modalities, to achieve a superior globe-preservation rate.

Considering the genetic *RB1* pathogenic variations in our population, we emphasize the 3 cases of unilateral hereditable retinoblastoma, 2 of which had a positive family history of retinoblastoma also presenting unilaterally in the affected progenitor. We believe that possibly these variations can be associated with a less aggressive retinoblastoma manifestation and better prognosis. We also highlight the case of a bilateral retinoblastoma associated with a *RB1* complete deletion that despite a good response to intra-arterial chemotherapy as presented with multiple recurrences during the study period, which we hypothesize being related with the specific large deletion. We hope that the awareness of the genetic profile of our population will increase our knowledge about possible prognostic factors linked to genotype and phenotype profiles. In Portuguese patients diagnosed in this study period we encountered a superior proportion of hereditable Retinoblastoma (53.6%) in comparison with the existent literature (approximately 40%)^6^, which can be explained by particular unknown genetic characteristic of our population, however we can not exclude a bias related to insufficient time of inclusion conditioning a small sample.

The incidence of retinoblastoma in Portugal appears to be similar to previous studies evaluating retinoblastoma incidence in Europe and in the United States of America. Year-of-birth controlled analysis presents less variability as the influence of age at diagnosis is attenuated and is, therefore, the method of choice for the incidence estimation.

Understanding the clinical characteristics of the Portuguese population will help the development of adequate strategies for screening the pediatric population. With our results we aim to improve awareness of this disease for medical care providers and pediatricians and to encourage them to perform screening procedures during clinical visits and, also, to educate parents for recognition of clinical signs of retinoblastoma. In the future, earlier diagnosis can be achieved, directly improving the prognosis of our population. New tools for early diagnosis are being developed and in process of validation, especially smartphone-based applications, that seems to improve early detection of Retinoblastoma and can be used not only by medical professionals but also parents and other caregivers.

## Conclusion

This is the first characterization of the Portuguese Population diagnosed with Retinoblastoma in the National Reference Center, during a period of 54 months. Incidence and demographic characteristics are in accordance with previous epidemiological studies from other populations. Although we still have a delay in the time of diagnosis, resulting in more than half of our patients with group D/E retinoblastomas at presentation, there is no major difference in the age at diagnosis comparing to other high-income countries.
